# Preliminary survey of a nemertean crab egg predator, *Carcinonemertes*, on its host crab, *Callinectes arcuatus* (Decapoda, Portunidae) from Golfo de Nicoya, Pacific Costa Rica

**DOI:** 10.3897/zookeys.457.6918

**Published:** 2014-11-25

**Authors:** Robert K. Okazaki, Ingo S. Wehrtmann

**Affiliations:** 1 Department of Zoology, Weber State University, Ogden, Utah, 84408-2505 USA; 2 Museo de Zoología, Escuela de Biología, Universidad de Costa Rica, 11501-2060 San José, COSTA RICA; 3 Unidad de Investigación Pesquera y Acuicultura (UNIP) of the Centro de Investigación en Ciencias Marinas y Limnología (CIMAR), Universidad de Costa Rica, 11501-2060 San José, COSTA RICA

**Keywords:** *Callinectes arcuatus*, egg mortality, crab egg predator, Central America

## Abstract

The possible presence of egg predators in brood masses of portunid crabs from Pacific Central America has not been studied yet. This survey reports the finding of a nemertean crab egg predator on the portunid crab, *Callinectes arcuatus*, from the Golfo de Nicoya, Pacific Costa Rica. Nemerteans were found in the egg masses of 26 out of the 74 crabs for a prevalence of 35%. The intensity (mean number of worms/ infected crab) was estimated to be 18 with a variance of 1–123 worms/infected crab. No nemerteans were observed either in the 19 *Callinectes arcuatus* from Golfo Dulce (southern Pacific coast) and the 10 *Portunus asper* from Herradura-Jaco (central Pacific coast). This nemertean is a member of the genus *Carcinonemertes*, which has been reported from the Caribbean coast of Panama. However, the encountered *Carcinonemertes* sp. is the first published finding and report from Costa Rica and Pacific Central America.

## Introduction

The arched portunid crab, *Callinectes arcuatus* Ordway, 1863 has become an increasingly important growing fishery resource along the Pacific coast from Mexico to Ecuador (Paul 1982; Castro and De Alteris 1989). In the Golfo de Nicoya of the Pacific coast of Costa Rica, Dittel (1993) reported a large population of this portunid crab. More recently, Fischer and Wolff (2006) analyzed the current level of exploitation of this resource by size frequency analysis of trap and trawl catches in the Golfo de Nicoya; the results of this study revealed the potential of increasing catch yield about 20% without a detriment to the crab population. Thus, the *Callinectes arcuatus* fishery is becoming recognized as a commercially important fishery for Costa Rica.

Phylum Nemertea, commonly called ribbon or proboscid worms, comprise approximately 1250 species (Kajihara et al. 2008). These ribbon worms are generally predators using their proboscis to capture prey. One monostiliferous hoplonemertean family, Carcinonemertidae, consists of nemerteans that are ecto-symbiont egg predators of decapods (Wickham and Kuris 1985, 1988, Kuris and Wickham 1987; Kuris 1993; McDermott 2006; Sadeghian and Santos 2010). These worms were implicated to cause 50% brood mortality in the *Cancer magister* fishery in northern California (Wickham 1979).

Carcinonemertids have been reported in Panamanian crabs, but so far only from the Caribbean coast (Collin et al. 2005). As far as we know, no published information is available about the possible infestation of egg masses of portunid crabs along the Pacific coast of Central America. Therefore, this study was conducted to determine if nemertean crab egg predators are present on *Callinectes arcuatus* from Golfo de Nicoya. If these nemerteans are found, then baseline numbers of infestation and incidence can be established to monitor future increases and their impact on a growing fishery.

## Methods

The following numbers and species of ovigerous crabs were collected by local fishermen: 74 specimens of *Callinectes arcuatus* from Golfo de Nicoya, central Pacific (10°0'00"N, 85°0'00"W), 19 of *Callinectes arcuatus* from Golfo Dulce, southern Pacific (8°32'16"N, 84°41'35"W), and 10 of *Portunus asper* (A. Milne-Edwards, 1861) from trawls off the coast of Herradura-Jaco, central Pacific (9°64'00"N, 84°65'00"W). All crabs were shipped under ice to the Universidad de Costa Rica in San José and then stored frozen.

After the crabs were allowed to thaw, eight pleopods of each crab were carefully snipped at the bases, wet weighed, and then placed in seawater. A MS5 Leitz dissecting microscope was used to examine the pleopods for nemerteans, worm sheaths, and worm egg strings. Sheaths without worms were considered in the count for infestation. During holding of the crabs in the containers by fishermen, stressed worms have been observed to leave the egg masses, which have become suboptimal for the nemerteans (J. Norenburg, pers. comm.). A total of 824 pleopods were analyzed.

To quantify the worm dispersion and infestation on host crabs, prevalence and intensity were measured. Prevalence, an indicator of infestation, was defined as numbers of infected host crabs per total host crabs × 100. Intensity, indicator of dispersion amongst infected host crabs, was defined as mean number of worms per infected host crab.

## Results

### Golfo de Nicoya

**Figure 1.**
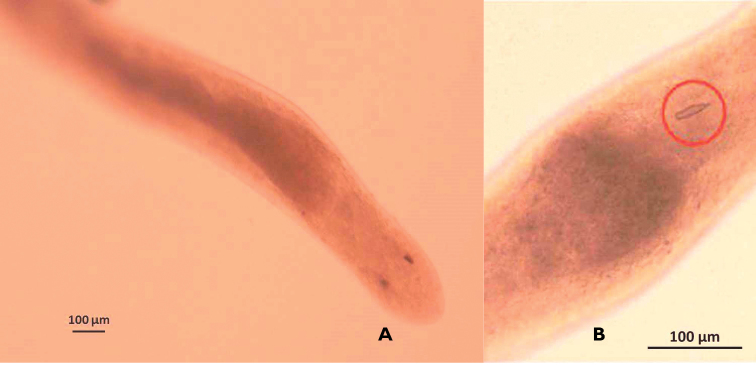
**A** Unidentified *Carcinonemertes* sp. from an ovigerous female of *Callinectes arcuatus* collected in Golfo de Nicoya, Pacific coast of Costa Rica. **B** Single basis and stylet observed in a nemertean from the egg mass of *Callinectes arcuatus* collected in Golfo de Nicoya, Pacific coast of Costa Rica.

A total of 26 egg masses out of 74 crabs were found to be infested with worms (Fig. 1A) for a prevalence of 35%. The worm was tentatively identified as belonging to the genus *Carcinonemertes* with a single stylet (Fig. 1B). Worm length averaged about ~7.0 mm with widths about 0.13 mm.

**Figure 2.**
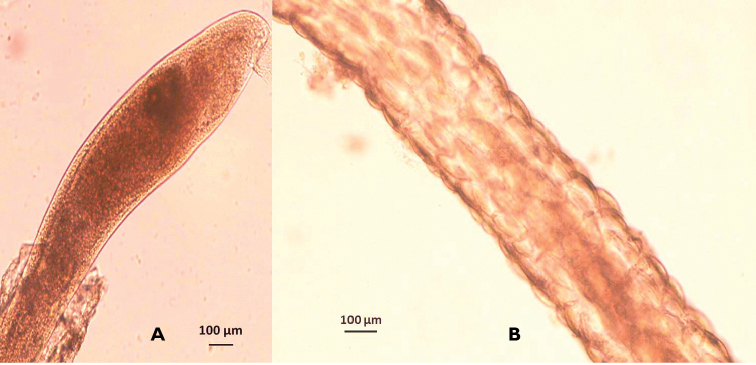
**A** Nemertean worm emerging from the mucous sheath, observed in the egg mass of *Callinectes arcuatus*, Golfo de Nicoya, Pacific Costa Rica. **B** Mucous sheath of a nemertean worm showing pronounced domed lapillae, observed in the egg mass of *Callinectes arcuatus*, Golfo de Nicoya, Pacific Costa Rica.

**Figure 3.**
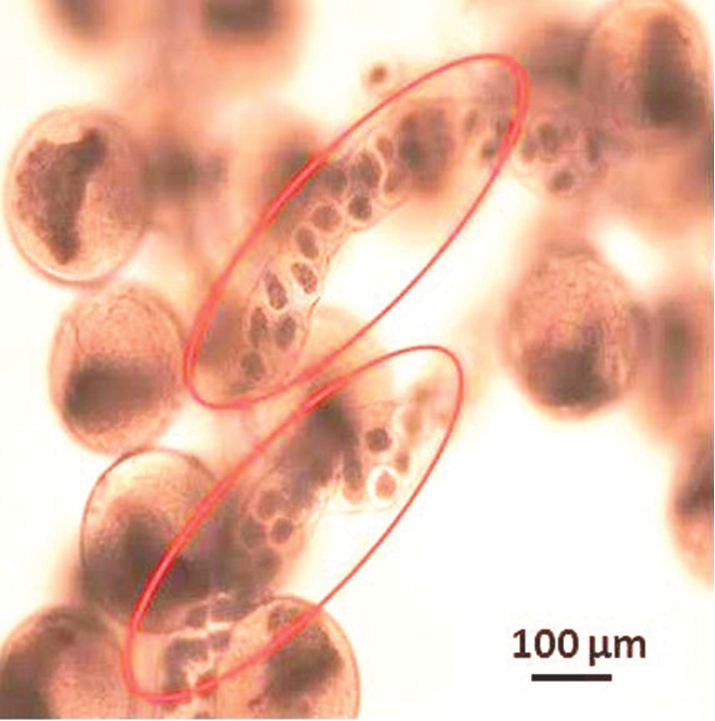
Nemertean worm egg strings (encircled) amongst eggs of *Callinectes arcuatus*, Golfo de Nicoya, Pacific Costa Rica.

Most worms were found to be inside sheaths (Fig. 2a). The mucous sheaths were linear, of small height (15–25 µm), and dome-shaped with uniformly distributed lapillae (Fig. 2b). Cursory investigation of the gills of the *Callinectes arcuatus* found no ensheathed nemerteans. Worm egg strings were also observed intertwined around the crab eggs (Fig. 3).

**Figure 4.**
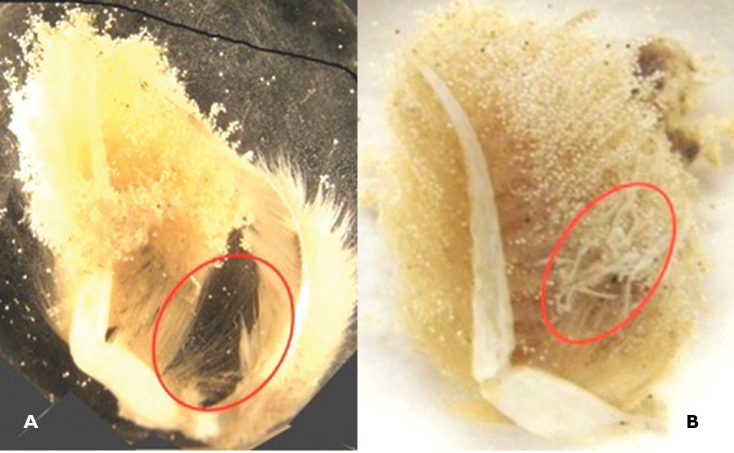
**A** Severe crab egg mortalities (encircled) at lateral base of a pleopod from an ovigerous female *Callinectes arcuatus*, Golfo de Nicoya, Pacific Costa Rica. **B** Nemertean worms (encircled) between the lateral middle and lateral base of a pleopod from an ovigerous female of *Callinectes arcuatus*, Golfo de Nicoya, Pacific Costa Rica.

Worms were found throughout the egg mass, but more often encountered at the base of pleopods. The pleopodal base of one crab showed pronounced egg mortalities (Fig. 4A) and an accumulation of worms in the mid pleopodal regions (Fig. 4B).

**Figure 5.**
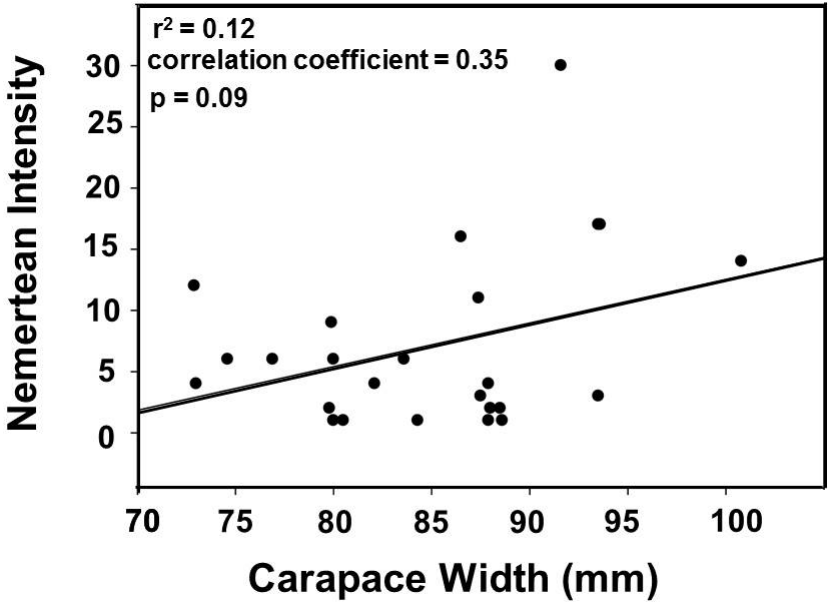
Linear regression analysis between size (carapace width) of ovigerous females of *Callinectes arcuatus*, Golfo de Nicoya, Pacific Costa Rica, and nemertean worm intensity.

Mean intensity (# worms/infested crab) was 18 (range 1–123 worms). Linear regression analysis showed no significance (r^2^ = 0.12; correlation coefficient 0.35; P = 0.09) between crab size (carapace width) and worm intensity (Fig. 5).

### Golfo Dulce and Herradura/Jaco

No worm infestations were found on crabs collected from Golfo Dulce and from off the coast of Herradura/Jaco.

## Discussion

This survey is the first report of a carcinonemertid crab egg predator on the portunid *Callinectes arcuatus* from Golfo de Nicoya in the Pacific coast of Costa Rica, and as far as we know, also the first published report from the Pacific coast of Central America. The observation of a single stylet near the brain is a characteristic of the genus *Carcinonemertes*. Further histological analysis will be needed to confirm the presence of the Takakura’s duct system (Humes 1942). In male *Carcinonemertes*, this duct system includes the vas efferens, vas deferens, seminal vesicle and gonoduct (Shields and Kuris 1990).

Nemerteans have been previously reported on the rafting crab, *Plagusia immaculata* Lamarck, 1818, from Pacific Panamanian coast (M. Torchin, unpubl.) and on unidentified crabs from Bocas del Toro, Caribbean coast of Panama (Collin et al. 2005). From unpublished 2004 survey of Panamanian crabs from the Caribbean coast (C. Santos, pers. comm.) carcinonemertids have been reported on *Leptodius floridanus* (Gibbes, 1850), *Macrocoeloma trispinosum* (Latreille, 1825), *Mithraculus cinctimanus* (Stimpson, 1860), *Mithraculus forceps* (A. Milne-Edwards, 1875), *Mithrax caribbaeus* (M. J. Rathbun, 1900, *Mithrax spinosissimus* (Lamarck, 1818), *Panopeus lacustris* Desbonne, 1867 and *Pilumnus pannosus* M. J. Rathbun, 1896.

This study found a 35% prevalence of this carcinonemertid on *Callinectes arcuatus* from the population from Golfo de Nicoya and none from Golfo Dulce. Wickham (1979) reported 100% prevalence for *Carcinonemertes errans* on Dungeness crab, *Cancer magister* (Dana, 1852) while Shields et al. (1990) found >97% prevalence on the yellow rock crab, *Cancer anthonyi* (M. J. Rathbun, 1897). Golfo de Nicoya is considered one of the most productive estuaries in the world (Cordoba-Muñoz 1998; Gocke et al. 2001), and is also the most important fishery area in Costa Rica (Cortés and Wehrtmann 2009). The Golfo de Nicoya is highly impacted by human activities and one of the most polluted regions along the Pacific coast of Costa Rica (Vargas 1995). These conditions may enhance the prevalence and transmission of these nemertean crab egg predators. In contrast, Golfo Dulce is a deep tropical, highly stratified and low-productivity fjord (Quesada-Alpízar and Cortés 2006). Especially the limited water circulation in this tropical fjord may restrict dispersion and transmission of nemerteans. Kuris et al. (1991) reported that infestation of nemerteans on the red king crab was highly variable; high egg mortalities were observed in geographically clustered crab populations.

The carcinonemertid encountered in our study may be host-specific to *Callinectes arcuatus* and have not exploited other crab hosts, such as *Portunus asper* from offshore of Herradura and Jaco. Further investigations are needed to determine the extent of nemertean prevalence on other potential host crabs.

In this study, the estimated infestation of 18 is low compared to 46,000 reported for *Carcinonemertes errans* Wickham, 1978 on *Cancer magister* by Wickham (1979) and 692 (86.5 worms/pleopod × 8) for *Cancer anthonyi* (Shields et al. 1990). This low intensity may be a reflection of several factors. Firstly, this symbiotic relationship between the worm and *Callinectes arcuatus* may have evolved most recently. Secondly, the artisan fishery inclusion of small-sized crabs, as well as both males and females, might contribute to the low reproduction of worms, which need ovigerous crabs to complete their reproductive cycle. Therefore, the lower intensities observed in *Callinectes arcuatus* and *Cancer anthonyi* compared to *Cancer magister* could be due to the fact that both female and male crabs are taken out in the *Callinectes arcuatus* and *Cancer anthonyi* fisheries.

Although in this preliminary study, worm incidence was low for *Callinectes arcuatus* from the Golfo de Nicoya, future monitoring of the carcinonemertid is strongly recommended. Collapse of this growing artisan fishery could potentially occur if worm population were somehow to explode, especially in these times of unstable climatic change. In 1981, the red king crab fishery recorded the third highest historical yield; however three years later, the fishery suffered the lowest catch in its 30 year history (Kuris et al. 1991). High red king crab egg mortality was correlated with high-intensity infestations of nemerteans in the egg mass (Kuris et al. 1991).

In the present study, the worms and their sheaths were observed in the medial middle and base areas of the pleopod. Pleopods of one crab sample showed very high egg moralities in these regions (refer to Fig. 4A, B). These findings are in agreement with similar observations from other crabs. Higher worm presence and abundance as well as egg mortalities in these pleopodal areas were also reported for *Cancer anthonyi* (Shields et al. 1990) and for *Cancer magister* (Okazaki and Kuris 2004).

Most spawning of *Callinectes arcuatus* occurs during the dry season from December to April, when ovigerous females migrate from the low salinity areas of the inner gulf to the high salinity waters in the outer gulf where the larvae hatch (DeVries et al. 1983). Larval development is completed within 70 days (Paul 1982; Dittel and Epifano 1984). Further studies are suggested to determine whether nemerteans complete their life cycle on one brood of host eggs or remain on the crab for a subsequent brood to complete another reproductive cycle. After host eclosion, carcinonemertids on portunid crabs encyst between the branchial lamellae and then lie dormant until the female crab oviposits (Humes 1942; Shields and Kuris 1990). In this study examination of branchial chambers of female crabs did not reveal encysted worms but barnacles from the genus *Octolasmis* (unpubl observations).

In this study, frozen crab samples were analyzed due to the logistics and limitations of receiving ovigerous crabs from fishermen. Worms have been observed to leave the crab egg mass when the crabs are out of the water (J. Norenburg, pers. comm.). Future studies using freshly trapped ovigerous crabs are suggested to improve infestation and incidence numbers. Also live nemerteans would allow for further histological and DNA analyses for phylogenetic relationships with other *Carcinonemertes* species.
